# Financial inclusion and monetary policy effectiveness: A sustainable development approach of developed and under-developed countries

**DOI:** 10.1371/journal.pone.0261337

**Published:** 2021-12-22

**Authors:** Muhammad Usman Arshad, Zeeshan Ahmed, Ayesha Ramzan, Muhammad Nadir Shabbir, Zahid Bashir, Fahad Najeeb Khan

**Affiliations:** 1 Department of Commerce, University of Gujrat- Hafiz Hayat Campus, Gujrat, Pakistan; 2 Department of Management Sciences, University of Lahore, Gujrat, Pakistan; 3 Department of Economics and Finance, Pakistan Institute of Development Economics, Islamabad, Pakistan; 4 School of International Trade and Economics, Central University of Finance and Economics, Beijing, China; 5 Noon Business School, University of Sargodha, Sargodha, Pakistan; Universidad de Castilla-La Mancha, SPAIN

## Abstract

The study explores the causal relationship between monetary policy effectiveness and financial inclusion in developed and under-developed countries. Structural Vector Auto-regressive techniques have been inducted to explore the relationship between monetary policy effectiveness and financial inclusion. The study covers the secondary data of 10 developed and 30 underdeveloped countries throughout 2004–2018. It is concluded that monetary policy effectiveness and financial inclusion do not have a contemporaneous impact on each other. Nevertheless, the reduced-form Vector Auto-regressive witness the reverse causality between financial inclusion and monetary policy effectiveness in developed countries. Thus, effective monetary policy enhances financial inclusion in a country, and a higher degree of financial inclusion lowers the inflation rate and makes monetary policy effective. One way causality from monetary policy effectiveness to financial inclusion can be observed in under-developed countries. Using the Structural Vector auto-regressive technique and financial inclusion index composed of three-dimension to examine the relationship of monetary policy effectiveness and financial inclusion in developed and developing countries is considered the study’s significant contribution.

## 1. Introduction

Financial inclusion has become an intriguing issue of the 21st century, and it is still creating a significant gap between the under-developed and developed nations. According to the Global Findex report, almost all unbanked adults live in under-developed countries. Nearly half of them came from seven countries: Bangladesh, China, Pakistan, Indonesia, Mexico, India, and Nigeria. The emphasis of both the world’s policymakers, academics, and professionals is maximum financial inclusiveness. An inclusive financial system provides equal opportunities to individuals and businesses to get an easy and affordable financial product and services to meet their needs, i.e., transaction, payments, saving, and credit [1]. It has been transforming impacts at initial levels such as spread awareness, improves financial knowledge, provides investment opportunities, helps people smoothen their consumption against shocks, and empower women. All these initial impacts lead to a higher economic outcome, i.e., escaping poverty, reduce income inequality, and set the economy on the path of development.

Economic growth, low levels of unemployment, and a stable inflation rate are the primary goal of any country’s central bank. These goals can only be achieved if the monetary policy works effectively in the economy. Monetary authorities of any country endorse monetary policy by managing interest rate or money supply in the economy to achieve specific objectives, i.e., price stability, promoting economic growth, financial stability, and inflation control. These specific goals can only be achieved if monetary policy actions are transferred effectively in the economy. There are different transmission mechanisms through which the policy actions are transferred into the economy. This monetary transmission mechanism connects the financial system to monetary theory [2]. The higher level of financial inclusion increases the pace and coverage of transmission in the financial system and makes monetary policy more effective. The available literature shows that monetary policy effectiveness has a significant and causal relation to financial inclusion. [3] infer that a higher degree of financial inclusion improves monetary policy effectiveness. Financial inclusion makes it possible to extend the monetary policy to financially exclude and helps policymaker’s better to forecast inflationary movement [4]. [5] analyze an inverse but significant relation between financial inclusion and inflation in SAARC countries. [6] examine the bi-causal link between financial inclusion and monetary policy. An increase in access to financial products and services increases investment expenditure, increases aggregate demand, and increasing interest elasticity, making monetary policy more sensitive. At the same time, monetary policy instruments like changes in the lending rate and saving rate promote financial inclusion.

The nexus of financial inclusion and monetary policy in the studies, as mentioned earlier, has been done in the case of underdeveloped countries but does this relationship stays the same in developed nations? This study addresses this gap by taking developed and under-developed countries. The inflation rate is used as a proxy of monetary policy effectiveness as it depicts monetary policy success [3, 7, 8]. The study covers the annual data from 2004 to 2018 using the Structural Vector Autoregressive (SVAR) technique. To measure financial inclusion, a multidimensional index is constructed based on Principle Component Analysis (PCA) using the access (supply side), usage (demand side), and barriers (distance, lack of trust, documentation, and affordability). There is a considerable gap in the levels of financial inclusion in developed and underdeveloped countries. Under-developed countries face different demand-side or supply-side barriers. These barriers highlight the gap that why under-developed countries face a lower level of financial inclusion.

Furthermore, the individuals of developing states face involuntary financial exclusion. Financial exclusion builds a sizeable informal sector in the economy. So, whenever the central bank announces the monetary policy, the financial decision taken by households and small businesses (informal sector) remains unaffected by policy actions and tends to hamper the transmission of the monetary policy. By removing barriers, more people can be included financially, enhancing the monetary policy effectiveness [9].

Financial inclusion can improve the monetary transmission channels and helps policymakers to make better decisions while targeting any one or both of them. The previous studies demonstrated no relationship between financial inclusion and monetary policy effectiveness in developed countries. The study contributes to the discipline of economics in several aspects. First, the study composes a financial inclusion index based on three dimensions such as access, usage, and barriers. Second, the study uses the Structural Vector Autoregressive technique to examine the causal relationship between financial inclusion and monetary policy effectiveness, unlike other studies [3, 6–8]. Structural VAR is used to capture the contemporaneous interdependence among variables by imposing certain restrictions based on economic theory. Lastly, to the best of our knowledge, the study is the first to empirically investigate the relationship between monetary policy effectiveness and financial inclusion in developed and under-developed countries; furthermore, how these nexuses operate in the economies of those countries at different development levels. The study is organized as follow: Section 2 review all the existing literature. Section 3 explains the data and methodology. Results are discussed in section 4. Section 5 presents the conclusion and policy recommendations.

## 2. Literature review

### 2.1 Theoretical literature

Monetary policy is a powerful tool for a central bank to achieve its price stability, economic growth, financial stability, and a high level of employment. We consider two aspects that show how monetary policy depends upon the level of financial inclusiveness. First, the policymaker faces a tradeoff between inflation and output volatility [10]. Secondly, individual’s access to savings and credit through the financial system helps them smoothen their consumption in a period of fluctuations. With the lower degree of financial inclusion, policymakers should reduce output volatility as individuals are incapable of sustaining spending as income reduces. So, in that case, policymakers give more weightage to stabilize the output rather than stabilize inflation.

Monetary policy affects the economy through different mechanisms. These transmission mechanisms include the interest-rate effect, exchange rate effect, asset price effect, and credit channel [11]. According to the conventional interest rate channel, a natural interest rate cut reduces the cost of capital. These lower interest rates (real) then lead to an increase in business, residential and inventory investment, and durable consumer expenditure, raising the aggregate output. This infers that targeting interest rates to achieve the objective of the monetary policy would be ineffective with a lower level of financial inclusion. The credit view is another essential monetary transmission channel. It is associated with problems in financial markets. The credit view is further subdivided into two different channels of monetary transmission, i.e., the bank lending channel and the balance sheet channel. In the balance sheet channel, any alterations in monetary policy affect the borrower’s balance sheets and income statements. The bank lending channels focus more on providing loans by institutions [12]. An expansionary monetary policy causes the money supply to rise, leading to more loans at lower borrowing costs due to increasing bank reserves. As households and small businesses rely on these loans to finance their activities, this loan expansion causes investment spending to rise [2]. Thus, lower borrowing cost tends to increase the financial inclusion.

Transmission of monetary policy via the banking lending channel is a deeply-rooted phenomenon in economics/banking literature [13]. This argument is supported by [7], who used a credit view transmission channel to discover the nexus between monetary policy and financial development. Monetary policy transmission channel is also influenced by individual financial health as it determines their degree of access to credit and the condition under which it is granted to them [14]. The extravagant access to credit increases the risk of bad debts that can cause financial instability and lead to inflation [4]. So, increasing financial inclusion must be effective for the economy and financial system.

The strength of any transmission channel depends upon the state of the financial system. The outcome of any monetary policy in an underdeveloped financial system takes a relatively shorter time to disappear from the economy altogether. However, the enhanced banking system might help improve the monetary policy mechanism [15]. [16] also draw attention to the banking condition to have effective transmission, promoting financial and monetary stability. [17] documented the effect of monetary policy changes as bank capital and liquidity change. More capitalized banks significantly transfer changes in monetary policy in comparison to lower capitalized banks. A sound banking system mobilizes small and scattered saving of community and make them available for investment in productive enterprise and plays a substantial role in accelerating the financial inclusion.

Financial inclusion can be an instrument for generating monetary fuel to achieve inclusive economic growth. In a broader context, it promotes economic inclusion by improving the living condition of poor people with better facilities and generate employment opportunities. A higher disposable income leads to more significant savings and a broader deposit base for financial institutions [9]. Greater financial inclusion enables monetary transmission channels to work more efficiently as it increases the share of the formal sector. A large informal sector negatively affects the monetary transmission mechanism as the financial decision taken by financially excluded are not affected by monetary policy [9]. Thus, the inclusion of such persons in the overall financial system could result in positive externalities, enabling monetary policy to be more effective. Similarly, monetary policy instruments such as changes in the lending rate and saving rate promote financial inclusion [6].

### 2.2 Empirical literature

The concept of financial inclusion has gained attention in the 21st century. The early literature defines the inclusive financial system and its importance. Over time, the researchers focus on the determinants of financial inclusion [18–20]. Fewer studies examine the impact of financial inclusion on economic growth, income disparities, and poverty [21–23]. However, there is confined literature available on financial integration that affects the monetary policy. [24] applied the SVAR approach on quarterly data and concluded that monetary policy transmission is weak because of the underdeveloped financial sector. However, monetary policy still affects inflation and output.

A contractionary monetary policy decreases output and inflation, but there is no effect on the exchange rate. Monetary policy has no significant effect on private sector credit even though private sector credit innovation raised inflation. [3] compute the effect on monetary policy effectiveness due to financial inclusion and concluded that a higher level of financial inclusion helps conduct the monetary policy effectively. Greater financial inclusion indicates that many people are involved in formal saving and investment, making monetary policy rates more sensitive. The model does not give powerful results of the association between financial inclusion and monetary policy and lacks theoretical backup. [25] infers that the monetary policy and bank lending transmission channel have no significant effect on aggregate demand. However, the optimal monetary policy is sensitive to the degree of financial inclusion, and the degree of financial inclusion is correlated to the level of autonomy of the central bank [10]. In other words, a central bank that is highly autonomous in its monetary policy decision is more likely to carry out optimal monetary policy.

A higher degree of financial inclusion is beneficial to maintain monetary stability [26]. Firstly, it helps in smoothing the consumption in response to any economic shock and encourages the consumer to save through a financial institution. Secondly, in greater financial inclusion, the interest rate is proved to be an effective transmission mechanism channel, and lastly, increased financial inclusion fosters financial stability. [4] analyzed financial inclusion influence effectiveness of monetary through VAR technique. The author considers inflation as a policy instrument of monetary policy effectiveness. In the short run, only 30% of the variation in the inflation rate is explained by financial inclusion. However, 70% variation is estimated in the long run. The overall results show that financial inclusion does impact the effectiveness of the monetary policy.

Instead of taking few indicators to measure financial inclusion, [5] formed an index and explained how financial inclusion influences monetary policy in the SAARC region. Demographic, geographic, and banking penetration construct the financial inclusion index through the principal component analysis (PCA) method. PCAs show that Maldives is placed first in the financial inclusion among all SAARC regions, and Pakistan is facing an extreme financial exclusion with the negative index. The results imply the significant negative relation of financial inclusion with inflation. Financial accessibility may reduce inflation and bring price stability to the economy.

[27] highlight two implications for the monetary policy under different levels of financial inclusion. First, they evaluate how emerging Asian economies’ prices and output respond against interest rate shocks under different degrees of financial inclusion. The result infers that output and prices are more interest-sensitive in economies where people are more financially included. That is because of the interest rate’s role in consumption. A higher interest rate reduces the consumption demand of financially included individuals. This results in the reduction of real wages and consumption demand of financially excluded. Secondly, the central bank targets headline inflation instead of core inflation when there is a higher share of food in the total consumption expenditure and a lower degree of financial inclusion.

Monetary policy effectiveness granger cause financial inclusion [8]. This means that financial inclusion does not significantly affect monetary policy effectiveness, but the monetary policy does. To achieve a higher degree of financial inclusion, heightened monetary policy is required. The impulse response results show that the shocks in financial inclusion, interest rate, and money supply explain the variation in monetary policy in the short run. However, only interest rates explain more than 45% variation in monetary policy in the long run. [7] observed an inverse relationship between financial development and monetary policy effectiveness. Besides, the study discovers that the monetary policy is less effective in the bank-oriented financial system than a market-oriented financial system. In the developing economies, which experienced more financial development, the monetary policy’s impact on output becomes lessen, while in advanced economies, it strengthens the impact on inflation by monetary policy. A higher level of financial inclusion lowers the inflation rate [28].

[29] adopted a panel vector error correction model to test the hypothesis that monetary policy transmission is less effective in economies with a lower degree of financial inclusion than ones with an increased degree of financial inclusion. It is illustrated that the countries with more developed financial systems can transmit monetary policy effectively. Higher financial inclusion, a well-structured economy, and a financial system allow policymakers to control monetary policy transmission more effectively without any leakages [30]. A well-developed banking system can see the multiplier effect of monetary policy and increase bank liquidity. Net national income is considered an essential factor in the relationship between monetary policy and financial inclusion. Inflation response negatively to the financial inclusion index, money supply, and net national income and positively to interest rate [31].

[32] highlights three ways through which a higher level of financial inclusion impacts monetary policy choices. First, the higher degree of financial inclusion may bound the central bank to emphasize core inflation more than headline inflation. Second, a higher degree of financial inclusion made the interest rate an effective tool of monetary policy. Financial inclusion provides means to entrepreneurs to fund investments other than retained earing, and a reduction in policy rate stimulates borrowing demand. Thirdly, the central bank may want to modify its interest rate rule to guarantee determinacy and optimality. Optimal policy focuses more on inflation stabilization instead of output stabilization.

The growth effect of monetary policy in advanced economies is more pronounced due to a robust financial structure than in developing economies. It is most noticeable in the market-concentrated financial system compared to the bank- concentrated financial system [33]. [6] used the inflation rate as a monetary policy to check the effectiveness of the monetary policy. The monetary policy rate is a better measure of monetary policy effectiveness as inflation is just an outcome variable. To measure financial inclusion, an index is formed using demand-side and supply-side factors. The results imply a bi-directional relation of financial inclusion with monetary policy. Lags of GDP growth rate and the inflation rate have a significant impact on financial inclusion. Variance decomposition shows that any shock in monetary policy significantly influences the variation of financial inclusion in the long run and vice versa.

Monetary policy positively affects financial inclusion that gradually fades away with time [34]. Effective monetary policy can lead to the sustainable growth of financial inclusion. However, at the same time economic fundamental has an inverse effect on financial inclusion. The regional imbalance of economic development is the main obstacle to the sustainable growth of financial inclusion. The effectiveness of monetary policy is dependent upon the transmission channel. Credit channel and liquidity channel transmit any monetary policy actions in the financial system. In economies with a less developed financial system, the credit channel dominates the liquidity channel. Countries with less-developed financial systems experienced higher lending rates and asymmetric behavior [35]. Monetary policy experienced a weak bank lending channel in developing countries using aggregate data. Unanticipated monetary policy changes influence the loan application, loan volumes, and loan rates [17]. A contractionary monetary policy leads to a reduction in the bank supply of loans to the firms. The banks with low capital are affected more by tight credit conditions and higher loan rates than more capitalized banks. The literature on the relationship between financial inclusion and monetary policy effectiveness is still growing. Many kinds of research have attempted to examine the relationship in a single country [3, 4, 28, 34] or a group of regional countries SAARC region [5, 6, 8, 29, 33].

## 3. Data and methodology

### 3.1 Data sources and variable

The study covered the secondary data of 10 developed The World Economic Situation and Prospects classified all the countries. The countries are taken from https://www.un.org/en/development/desa/policy/wesp/wesp_current/2014wesp_country_classification.pd and 30 underdeveloped countries throughout 2004–2018. These countries are selected subject to data availability and are true representative of population. Table 1 represent the detailed list of developed and under developed countries. The data is obtained from various sources and the description about the variables and sources is given in (Table 2). The data has been focused largely on the emergence of relationship between monetary policy effectiveness and financial inclusion in developed and under-developed countries. The study emphasize on the development of a sound conclusion in relarion to the hypothesis. The inflation rate (Consumer Price Index) is used as a proxy for monetary policy effectiveness. The data related to inflation rate and other controlled variables i.e. official exchange rate, broad money (M2), interest rate, and economic growth (GDP growth rate) are gathered from World Development Indicator (WDI). The data of demand-side (usage) and supply (access) side factors of financial inclusion are taken from the Financial Access Survey (FAS) IMF data. World Bank Global Findex conducted a survey of about 150,000 adults regarding the use of the financial product and summarized the data of 180+ financial inclusion indicators. The data in relation to barriers (distance, trust, affordability, documentation) is obtained from the Global Findex of 2011, 2014, and 2017. The study used the compound average growth rate to find out missing values.

**Table 1:** List of countries.

Developed countries	Under-developed countries		
Australia	Algeria	Philippines	Mauritius
Austria	Armenia	Ukraine	Peru
Belgium	Bangladesh	Malaysia	South Africa
Chile	Costa Rica	Nicaragua	Thailand
Czech Republic	Honduras	Mexico	Turkey
Hungary	India	Mozambique	Namibia
Latvia	Indonesia	Uganda	North Macedonia
Seychelles	Kenya	Argentina	Georgia
New Zealand	Rwanda	Ecuador	Moldova
United State	Pakistan	Jamaica	Madagascar

**Table 2:** Data and variable description.

variables	Notation	Description	Data Source
**Inflation rate**	INFRA	Inflation, consumer prices (annual %)	WDI
**Financial inclusion index**	**FII**	**Index consisted of 10 variables**	
1	DCB	Outstanding deposits with commercial banks (% of GDP)	FAS, IMF data
2	LCB	Outstanding loans from commercial banks (% of GDP)	
3	CBBPKM	Number of commercial bank branches per 1,000 km^2^	
4	CBP	Number of commercial bank branches per 100,000 adults	
5	ATMSKM	Number of ATMs per 1,000 km^2^	
6	ATMSAD	Number of ATMs per 100,000 adults	
7	DISTANCE	No account because financial institutions are too far away (% age 15+)	Global Findex, World Bank
8	DOCUMENTATION	No account because of lack of necessary documentation (% age 15+)	
9	TRUST	No account because of lack of trust in financial institutions (% age 15+)	
10	AFFORDABILITY	No account because financial services are too expensive (% age 15+)	
**Board money**	M2	Broad money (% of GDP)	WDI
**Economic growth**	GDP	GDP growth (annual %)	
**Interest rate**	IR	Lending interest rate (%)	
**Exchange rate**	ER	Official exchange rate (LCU per US$, period average)	

### 3.2 Construction of financial inclusion index

Financial inclusion cannot be measured with a single variable as one variable cannot accurately depict. So, there are different indicators used to measure financial inclusion. The most commonly used indicators by different studies are the banking population, geographic penetration, availability of financial services to the people, and usage of the financial services [6, 18, 20, 28, 31, 36].

In 2004, the IMF financial access survey listed the indicators under two broad categories (i) usage and (ii) access indicators. The usage or demand-side evaluates the extent to which people use financial services. The indicators lie in the usage category are (a) Outstanding loans from commercial banks, (b) Outstanding deposits from commercial banks, (c) Numbers of deposit accounts with commercial banks per 1000 adults (d) Number of borrowers from the commercial bank per 1000 adults. Access estimates the supply of financial services. The indicator is (a) the Numbers of commercial bank branches per 1000*km*^2^ (b)Numbers of commercial bank branches per 1000 adults (c) Number of ATMs per 1000*km*^2^ (d) Number of ATMs per 1000 adults.

This indicator does not explain why some people are financially exclusive despite capturing the demand and supply side. In 2011, the global Findex design a questionnaire that highlights the significant barriers of individuals who do not own a bank account. The reported barriers include distance (banks are too far away), affordability (expensive financial services), documentation (does not have necessary documents), trust (people do not trust their financial institution), family members already own an account.

The following are the variables we are using to compute an index.

1. **Demand-side factors (usage)**
  - Outstanding loans from commercial banks (LCB)
  - Outstanding deposits from commercial banks (DCB)
2. **Supply-side factors (access)**
  - Numbers of commercial bank branches per 1000*km*^2^ (CBBPKM)
  - Numbers of commercial bank branches per 1000 adults (CBBPA)
  - Number of ATMs per 1000*km*^2^ (ATMSKM)
  - Number of ATMs per 1000 adults (ATMSAD)
3. **Barriers**
  - Distance
  - Documentation
  - Affordability
  - Trust

The Principal Component Analysis (PCA) technique is used to handle the suspected multi-collinearity among variables to construct a financial inclusion index. [18] and [37] also used a Principal Component Analysis approach to construct a financial inclusion index. PCA transforms the correlated variable into orthogonal and uncorrelated variables (factors or pcs) by reducing correlated variables while keeping as much information as possible in the variables. These factors are arranged so that the first few values retain the majority of variation in all the original variables [38]. According to this method, the index of *the j*^*th*^ factor can be addressed as

$${FII}_{J}=W_{J1}X_{1}+W_{J2}X_{2}+\cdots +W_{JP}X_{P}$$ (1)

Where FII is the financial inclusion index, *W*_*j*_ is the factor of coefficient’s weight; X is the original variable; p is the number of variables in the equation.

A separate index has been formed for both developed and under-developed countries. In developed countries, only two dimensions are used for index construction, i.e., demand and supply side, but barriers are also incorporated in underdeveloped countries.

The financial inclusion index for developed countries is composed of six indicators. The index can be specified as follow:

FII=f(LCB,DCB,CBBPKM,CBBPA,ATMSKM,ATMSAD) (2)

For underdeveloped countries, the index is made up of ten factors.


FII=f(LCB,DCB,CBBPKM,CBBPA,ATMSKM,ATMSAD,DISTANCE,,AFFORDABILITY,TRUST,DOCUMENTATION) (3)

### 3.3 Model specification

The study employed a panel Structural Vector Autoregressive (SVAR) technique for estimations. The SVAR model imposes restrictions based on economic theory is the only difference between VAR and SVAR. The VAR model is first suggested by [39]. He criticized the model because of the solid restrictions and assumptions about the dynamic nature of the relationship between macro-economic models and proposed VARs as an alternative model. These multivariate time series models are designed to capture the joint dynamics of multiple time series, forecasting macroeconomic variables and structural analysis. The variables in the VARs system are considered endogenous and depend on its lags, lags of all other variables [37].

#### 3.3.1 SVAR model

The generalized panel SVAR model can be written as follow:

$$BY_{it}=\Gamma_{0}+\Gamma_{it}Y_{it-k}+\varepsilon_{it}$$ (4)

B is a square matrix that captures the contemporaneous effect of variables. *Y*_*it*_ is a vector of the K endogenous variable. The subscript *i* = 1….N and *t* = 1…. T represent country and time. *ε*_*it*_ is the structural shock. The structural shock must satisfy the following conditions (i) E (ε_*t*_) = 0, (ii) $\text{E}(\varepsilon_{t}\varepsilon_{s}^{\prime })=\Sigma_{t}$ and (iii) $\text{E}(\varepsilon_{t}\varepsilon_{s}^{\prime })=0$.

To obtain reduce form VAR equation, pre multiply *B*^−1^ with Eq (4)

$$Y_{it}=B^{-1}\Gamma_{0}+B^{-1}\Gamma_{it}Y_{it-k}+{B^{-1}\varepsilon }_{it}$$ (5)

Where *e*_*it*_ = *B*^−1^ ε_*it*_ is reduced from VAR errors that satisfy these conditions (i) E (*e*_*t*_) = 0 (ii) $\text{E}(e_{t}e_{t}^{\prime })=\sigma$ and (iii) $\text{E}(e_{t}e_{t-k}^{\prime })=0$.

The variance-covariance of the structural matrix is

$$\begin{array}{ll} \Sigma_{s} & =\text{E}(\varepsilon_{t}\varepsilon_{s}^{\prime })\\ & =\text{E}(Be_{t}e_{t}^{\prime }B\prime )=\text{EB}(e_{t}e_{t}^{\prime })B\prime \\ & =\text{B}\Sigma_{e}B\prime \end{array}$$

For the system to be exactly identified *n*^2^(*n*^2^−*n*)/2 restriction will be imposed to recover all the structural shocks from the reduced form VAR residual *e*_*t*_.

#### 3.3.2 Structural model

The study used a panel Structural Vector Autoregressive (SVAR) model to determine the causal link between financial inclusion and monetary policy effectiveness. Given the formula *n*^2^(*n*^2^−*n*)/2, the study applied 15 restrictions on the B matrix to identify the system. The matrix B estimate the contemporaneous impact of the variable on each other. The variables are list in order; the most exogenous ones are written before the endogenous ones. The diagonal shows that the coefficients are normalized to unity, and zeros represent the restrictions.


$$BY_{t}=\left[\begin{array}{cccccc} 1 & {Ø}_{12} & 0 & 0 & {Ø}_{15} & {Ø}_{16}\\ {Ø}_{21} & 1 & {Ø}_{23} & {Ø}_{24} & {Ø}_{25} & {Ø}_{26}\\ 0 & 0 & 1 & 0 & 0 & 0\\ 0 & {Ø}_{42} & 0 & 1 & {Ø}_{45} & {Ø}_{46}\\ 0 & {Ø}_{52} & 0 & {Ø}_{54} & 1 & {Ø}_{56}\\ 0 & 0 & 0 & {Ø}_{64} & 0 & 1 \end{array}\right]\left[\begin{array}{c} MS_{t}\\ IR_{t}\\ ER_{t}\\ FII_{t}\\ INF_{t}\\ EG_{t} \end{array}\right]$$ (6)

The first, second, third, and fourth zero restriction shows that exchange rate, financial inclusion inflation, and economic growth do not contemporaneous impact on the money supply. The fifth and sixth zero shows that interest rate does contemporaneously respond to exchange rate and economic growth. Money supply, financial inclusion, inflation, and economic growth do not affect the exchange rate in the same period. The study assumes that only interest rate, inflation, and economic growth have a contemporaneous impact on financial inclusion. The thirteenth and fourteenth restriction is imposed on the assumption that the exchange rate and economic growth does not have a contemporaneous effect on inflation. The last restriction is imposed that economic growth does not respond in the same period to the exchange rate.

#### 3.3.3 Reduced form VAR

The reduced form VAR (Eq 5) in matrix notation can be written as

$$\left[\begin{array}{c} MS_{t}\\ IR_{t}\\ ER_{t}\\ FII_{t}\\ INF_{t}\\ EG_{t} \end{array}\right]=\left[\begin{array}{c} {Ø}_{1}\\ {Ø}_{2}\\ {Ø}_{3}\\ {Ø}_{4}\\ {Ø}_{5}\\ {Ø}_{6} \end{array}\right]+\left[\begin{array}{cccccc} 1 & {Ø}_{12} & 0 & 0 & {Ø}_{15} & {Ø}_{16}\\ {Ø}_{21} & 1 & {Ø}_{23} & {Ø}_{24} & {Ø}_{25} & {Ø}_{26}\\ 0 & 0 & 1 & 0 & 0 & 0\\ 0 & {Ø}_{42} & 0 & 1 & {Ø}_{45} & {Ø}_{46}\\ 0 & {Ø}_{52} & 0 & {Ø}_{54} & 1 & {Ø}_{56}\\ 0 & 0 & 0 & {Ø}_{64} & 0 & 1 \end{array}\right]\left[\begin{array}{c} MS_{t-1}\\ IR_{t-1}\\ ER_{t-1}\\ FII_{t-1}\\ INF_{t-1}\\ EG_{t-1} \end{array}\right]+\left[\begin{array}{c} e_{1t}\\ e_{2t}\\ e_{3t}\\ e_{4t}\\ e_{5t}\\ e_{6t} \end{array}\right]$$ (7)

Due to limited data of barrier variables, the estimations are divided into three models. Model 1 estimates the relationship between financial inclusion and monetary policy effectiveness without incorporating barriers dimension in the composition of the financial inclusion index. In model 2, the financial inclusion index of under-developed countries incorporates the barrier’s dimension, whereas the developed countries index is made of only by taking access and usage indicators. Model 3 explores the relationship between financial inclusion and monetary policy throughout 2011–2018 with barriers data incorporating in the only index of under-developed countries.

Akaike information criterion, Hannan-Quinn information criterion (HQ), and Schwarz/Bayesian information criterion are used to choose autoregressive lag order. The properties of the structural PVAR model are typically summarized using impulse response function, Granger causality test, and decomposition of error variance forecasts. Granger causality test uses to predict future values of one variable using the past values of another variable. The impulse response functions explain the reaction of the dependent variable in the VAR system to shocks in the error term 𝜀𝑖𝑡 [38]. The impulse response function is derived from estimated VAR parameters and their standard errors, so it is necessary to estimate the confidence interval to get the impulse response function. The forecast error variance decomposition depicts the changes in the variables due to shocks in other variables and their shocks. It determines the severity of the total effect and provides the upcoming trends of variables when there is a shock in the economy.

## 4. Findings and discussions

### 4.1 Descriptive statistics

Table 3 shows the descriptive statistics of developed and under-developed countries, respectively. The mean values of deposits from commercial banks (DCB), loans from commercial banks (LCB), commercial bank branches per 1000km^2^ (CBBPKM), commercial bank branch per 1000 adults (CBBPA), the number of ATMs per 1000km^2^ (ATMSKM), and the number of ATMs per 1000 adults reveals a higher level of financial inclusion in developed countries in comparison to under-developed countries. Most people in under-developed countries prefer to borrow from friends and relatives rather than from financial institutions. This shows a lack of trust in financial institutions.

**Table 3:** Descriptive statistics.

Variables	DCB	LCB	CBBPKM	CBP	ATMSKM	ATMSPA	INF	MS	ER	IR	**EG**
**Developed Countries**											
** Mean**	56.844	65.554	28.196	28.332	65.507	89.231	3.0176	80.123	82.867	6.824	2.706
** Median**	57.270	51.172	10.605	29.582	45.952	69.368	2.301	79.613	1.453	6.184	2.570
** Maximum**	116.949	151.509	163.177	57.278	287.549	186.398	36.965	124.777	676.958	15.347	11.889
** Minimum**	27.250	21.897	0.643	11.286	2.925	31.728	‒2.405	33.867	0.629	1.471	‒14.402
** Std. Dev.**	21.773	35.667	40.828	11.819	76.635	47.729	4.249	20.902	175.686	2.702	3.105
**Observations**	150	150	150	150	150	150	150	150	150	150	150
**Under- Developed Countries**											
** Mean**	41.402	35.734	13.828	11.780	27.811	30.112	6.591	52.100	600.137	14.475	4.470
** Median**	36.363	31.319	7.587	10.252	15.271	23.601	5.561	48.396	33.950	13.211	4.791
** Maximum**	182.183	116.044	111.823	45.141	228.571	122.781	48.700	140.092	14236.94	60.000	13.866
** Minimum**	6.758	6.951	0.157	0.398	0.046	0.040	‒1.404	14.987	1.000	3.423	‒14.759
** Std. Dev.**	28.293	21.514	20.492	8.129	39.885	26.995	5.407	26.759	2016.640	8.539	3.325
** Observations**	448	448	448	448	448	448	448	448	448	448	448

**Note:** The table shows descriptive statistics of Developed and under-Developed countries. DCB, LCB, CBBPKM, CBBPA, ATMSKM, ATMSPA shows the financial inclusion situation of Developed under-Developed countries. The overall average of deposits and loans from the commercial bank (% of GDP) is relatively low in under-developed countries compared to developed countries.

The under-developed countries also experience a higher inflation rate (INF) and interest rate (IR) of average 6.6 and 14.5, while the developed sample has a mean value of 3 and 7. The mean of economic growth (EG) in developed countries is 2.7, but 4.5 in under-developed countries.

### 4.2. Model 1: Developed and under-developed countries analysis without incorporating barriers in an index

#### 4.2.1 Lag determination

Table 4 describes the lag determination statistics of developed and under-developed countries. The lag is determined on the minimum values of Akaike information criteria (AIC) and Hannan-Quinn information criteria (HQ), Schwarz information criteria (SC). Based on AIC, HQ, and SC, the study takes three lags of developed countries and under-developed countries.

**Table 4:** Lag determination.

Lag	Log L	LR	FPO	AIC	SC	HQ
**Developed countries**						
1	‒1524.64	1234.92	94661.55	28.48	29.52*	28.90
2	‒1467.33	101.07	64616.46	28.09	30.01	28.87
3	‒1410.72	93.66*	45067.12*	27.72*	30.52	28.86*
4	‒1379.38	48.44	50415.28	27.81	31.49	29.30
**Under-Developed countries**						
1	‒5530.06	4624.32	22948324	33.98	34.46*	34.17*
2	‒5481.51	93.24	21262647	33.90	34.80	34.26
3	‒5421.49	113.09*	18375233*	33.75*	35.07	34.28
4	‒5400.98	37.90	20215127	33.85	35.58	34.54

#### 4.2.2 Structural VAR

Tables 5 and 6 represent the contemporaneous effect of the coefficient of all variables in developed and under-developed countries, respectively. The results of the developed country show that only money supply, inflation, and economic growth have a contemporaneous impact (Table 5). In contrast, only the inflation coefficient is significant in under-developed countries (Table 6). In both economies, none of the variables have a contemporaneous effect on financial inclusion. The negative coefficients of both the inflation rate and financial inclusion indicate the inverse relationship between them.

**Table 5:** Estimation of the contemporaneous coefficient of developed countries.

MS	IR	ER	FII	INF	EG
1.000	‒0.050 (0.914)	0.000	0.000	0.382** (0.0024)	0.653** (0.001)
‒0.134 (0.129)	1.000	0.044** (0.017)	‒2.160 (0.112)	0.094 (0.507)	‒0.360 (0.169)
0.000	0.000	1.000	0.000	0.000	0.000
0.000	‒0.597 (0.996)	0.000	1.000	‒0.019 (1.000)	0.176 (0.998)
0.000	‒0.852 (0.999)	0.000	‒1.019 (0.99)	1.000	‒0.334 (0.999)
0.000	0.000	0.000	7.397 (0.128)	0.000	1.000

**Note:** Probability values in (), ** represent significance level at 5%.

**Table 6:** Estimation of the contemporaneous coefficient of under-developed countries.

MS	IR	ER	FII	INF	EG
1.000	‒0.028 (0.904)	0.000	0.000	0.249** (0.019)	0.114 (0.368)
‒0.139 (0.341)	1.000	0.002 (0.266)	‒4.601 (0.452)	‒0.408 (0.379)	0.422 (0.533)
0.000	0.000	1.000	0.000	0.000	0.000
0.000	‒34.799 (0.990)	0.000	1.000	‒20.537 (0.991)	‒9.629 (0.990)
0.000	‒2.452 (0.457)	0.000	‒17.841 (0.538)	1.000	1.377 (0.568)
0.000	0.000	0.000	6.691 (0.616)	0.000	1.000

**Note:** Probability values in (), ** represent significance level at 5%.

#### 4.2.3 Reduced-form vector autoregressive

The results related to developed and under-developed countries are shown in (Tables 7 and 8). The result indicates reverse causality between financial inclusion and inflation rate at a 10% significance level in developed countries. A higher level of financial inclusion means more people can take credits from financial institutions and increase the demand for loanable funds [5, 6, 29]. An increase in the policy rate increases the interest rate but reduces inflation, making monetary policy effective [11].

**Table 7:** Reduced-form VAR results of developed countries.

	FII	INF	ER	IR	MS	EG
**FII(-1)**	0.9049** [8.599]	0.1083 [0.141]	‒0.3562 [‒0.119]	‒0.0636 [‒0.239]	‒2.3089** [‒2.683]	1.3699** [2.403]
**FII(-2)**	‒0.3110** [‒2.180]	0.4274 [0.411]	‒0.7582 [‒0.188]	0.2125 [0.589]	2.3939** [2.050]	‒0.4439 [‒0.574]
**FII(-3)**	0.2053** [2.139]	‒1.2563*** [‒1.796]	4.1679 [1.533]	‒0.3771 [‒1.555]	‒0.0956 [‒0.122]	‒0.4589 [‒0.883]
**INF(-1)**	‒0.0254*** [‒1.789]	0.3746** [3.620]	0.3413 [0.849]	0.0558 [1.557]	‒0.2588** [‒2.229]	‒0.1321** [‒1.718]
**INF(-2)**	‒0.0031 [‒0.226]	‒0.4857** [‒4.824]	‒0.1490 [‒0.381]	‒0.0574 [‒1.644]	0.0798 [0.706]	0.2299** [3.071]
**INF(-3)**	‒0.0169 [‒1.325]	0.2267** [2.441]	0.1080 [0.299]	0.0023 [0.070]	‒0.3396** [‒3.258]	‒0.1278** [‒1.850]
**ER(-1)**	‒0.0029 [‒0.857]	0.0163 [0.658]	1.4114** [14.629]	‒0.0301 [‒3.497]	‒0.0306 [‒1.097]	0.0097 [0.526]
**ER(-2)**	0.0004 [0.068]	‒0.0183 [‒0.438]	‒0.6719*** [‒4.146]	0.0230 [1.590]	0.0031 [0.067]	‒0.0232 [‒0.749]
**ER(-3)**	0.0026 [0.697]	0.0725 [0.003]	0.2727** [2.626]	0.0068 [0.734]	0.0243 [0.809]	0.0145 [0.731]
**IR(-1)**	‒0.0077 [‒0.196]	‒0.1233 [‒0.430]	1.9029** [1.706]	0.5873** [5.903]	0.2807 [0.872]	‒0.3244 [‒1.520]
**IR(-2)**	0.01042 [0.227]	0.4325 [1.296]	‒2.1533** [‒1.659]	0.1177 [1.017]	‒0.3783 [‒1.010]	0.1327 [0.535]
**IR(-3)**	0.0234 [0.644]	‒0.1045 [‒0.395]	0.6593 [0.641]	0.2708*** [2.951]	0.2868 [0.965]	0.2378 [1.209]
**MS(-1)**	‒0.0074 [‒0.669]	‒0.2453** [‒3.044]	0.2738 [0.875]	0.0330 [1.181]	1.1614*** [12.845]	0.0134 [0.222]
**MS(-2)**	‒0.0075 [‒0.436]	‒0.0571 [‒0.458]	0.0210 [0.043]	‒0.0848*** [‒1.960]	‒0.2502** [‒1.787]	‒0.0631 [‒0.681]
**MS(-3)**	0.0113 [0.971]	0.2972** [3.508]	‒0.3184 [‒0.967]	0.0628** [2.138]	0.0383 [0.403]	0.0555 [0.882]
**EG(-1)**	‒0.0487*** [‒2.655]	0.1636 [1.223]	0.5368 [1.033]	0.0704 [1.519]	0.0821 [0.547]	0.4950*** [4.979]
**EG(-2)**	0.0162 [0.793]	0.1364 [0.919]	‒0.3924 [‒0.680]	0.0147 [0.286]	‒0.5122** [‒3.075]	‒0.1515 [‒1.3735]
**EG(-3)**	0.0074 [0.437]	0.2297*** [1.869]	‒0.0265 [‒0.055]	0.0741*** [1.740]	0.6782** [4.918]	‒0.0848 [‒0.928]
**R-squared**	0.735	0.578	0.996	0.866	0.973	0.399
**Adj. R-squared**	0.688	0.503	0.995	0.842	0.968	0.292
**F-statistic**	15.589	7.686	1243.355	36.266	200.805	3.724

**Note:** t-Statistics in [], *, **, *** represent significance level at 1%, 5% and 10%

**Table 8:** Reduced-form VAR results of under-developed countries.

	FII	INF	ER	IR	MS	EG
**FII(-1)**	0.8916** [53.572]	‒0.2211 [‒1.018]	18.7552** [2.596]	0.1201 [1.114]	‒0.0856 [‒0.538]	‒0.3887** [‒2.614]
**INF(-1)**	‒0.0058*** [‒1.703]	0.5736** [13.021]	‒0.3315 [‒0.226]	0.07174** [3.281]	‒0.0658** [‒2.040]	‒0.0975**[‒3.231]
**ER(-1)**	‒1.6836 [‒0.193]	‒7.5405 [‒0.664]	1.03581** [274.240]	1.4305 [0.254]	‒0.0002** [‒2.092]	0.0001 [1.317]
**IR(-1)**	0.0018 [0.784]	0.0389 [1.294]	0.8753 [0.875]	0.9732** [65.177]	0.0019 [0.087]	‒0.0072 [‒0.350]
**MS(-1)**	‒0.0003 [‒0.358]	‒0.0109 [‒1.158]	‒0.0655 [‒0.208]	‒0.0039 [‒0.833]	0.9858** [142.097]	‒0.0086 [‒1.322]
**EG(-1)**	0.0132*** [2.562]	0.0043 [0.064]	0.6046 [0.271]	0.0696** [2.087]	0.0840** [1.708]	0.3189** [6.935]
**R-squared**	0.881	0.359	0.995	0.936	0.985	0.183
**Adj. R-squared**	0.879	0.349	0.995	0.935	0.985	0.171
**F-statistic**	507.29	38.31	13259.45	998.35	4624.07	15.30

**Note**: t-Statistics in [], *, **, *** represent significance level at 1%, 5% and 10%.

In under-developed countries, the lag of the inflation rate has a significant negative impact on financial inclusion. Lower inflation promotes financial inclusion by changes in lending rate, saving rate, and changes in aggregate demand [8]. However, the lag of financial inclusion does not show any significant impact on the inflation rate. Under-developed countries witness a lower degree of financial inclusion, which creates an obstacle to transmit monetary policy action properly in the economy [9]. In the case of financial inclusion, the results of under-developed countries are the same as in Developed countries. The result coincides with the findings in the literature [6].

#### 4.2.4 Impulse response function

**Fig 1:**
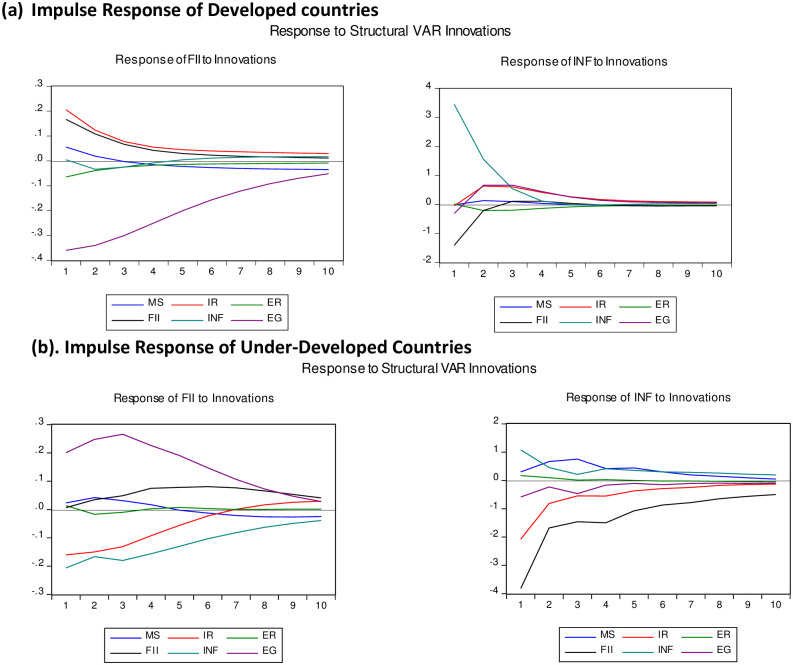
(a) Impulse response of developed countries. (b). Impulse response of under-developed countries.

The impulse response function represents the behavior of the variable over time due to random shock in another variable. The structural restrictions are employed to find more meaningful results. Fig 1(a) shows the response of financial inclusion and inflation to the shock in all endogenous variables of developed countries. The structural shock in inflation generates fluctuation in financial inclusion. The shock in inflation causes financial inclusion to decrease (panel a), and the response dies over time. Inflation’s negative response to the shock in financial (panel b) confirm the bidirectional causality.

In under-developed countries, financial inclusion responded negatively to the shock in inflation. In return, inflation also shows a solid response to the shock of financial inclusion. These findings support the negative relationship between financial inclusion and inflation. However, the VAR results in a highlight that only the lag of inflation has an impact on financial inclusion.

### 4.3 Model 2: Developed and under-developed countries analysis with barrier incorporating in an index

In this model, the under-developed financial inclusion index is based on access, usage, and barriers. In contrast, the financial inclusion index of developed countries is composed of taking only access and usage dimension due to the non-availability barriers data. The study uses the same results as model 1 for developed countries and compares them with under-developed countries in this model.

#### 4.3.1 Lag determination criteria

Table 4 model 1, three lags were taken based on AIC and HQ for developed countries. Table 9 shows the results of the lag order selection for under-developed countries. The SC and HQ criteria choose one lag, whereas AIC chooses three lags as most tests pick out the first lag, so we are taking one lag to estimate VAR/SVAR.

**Table 9:** Lag selection of under-developed countries.

Lag	Log L	LR	FPE	AIC	SC	HQ
0	‒7987.11	NA	59267136	48.74	48.81	48.77
1	‒5599.88	4672.56	35127619	34.40	34.89*	34.59*
2	‒5546.72	102.12	31643032	34.30	35.20	34.66
3	‒5497.74	92.27*	29251969*	34.22*	35.54	34.74
4	‒5471.55	48.393	31085558	34.28	36.01	34.97

#### 4.3.2 Structural VAR

Structural VAR shows the contemporaneous effect of all the variables in the system. A total of 15 restrictions are imposed based on theory.

1 and 3 out of 21 coefficients are significant at 5% in under-developed and developed countries, respectively Tables 5 and 10. In both economies, none of the variables have a contemporaneous effect on financial inclusion. The negative sign of the coefficient of financial inclusion and inflation indicates inverse relations but does not have a contemporaneous impact on each other.

**Table 10:** Estimation of the contemporaneous coefficient of under-developed countries.

MS	IR	ER	FII	INF	EG
1.000	‒0.0268 (0.907)	0.000	0.000	0.184** (0.000)	0.079 (0.593)
0.1803 (0.219)	1.000	0.002 (0.411)	‒6.809 (0.5185)	0.000	0.672 (0.547)
0.000	0.000	1.000	0.000	‒0.917 (0.612)	0.000
0.000	0.289 (0.994)	0.000	1.000	‒0.011 (0.999)	‒0.036 (0.997)
0.000	‒0.521 (0.999)	0.000	‒0.609 (0.999)	1.000	0.135 (0.998)
0.000	0.000	0.000	4.050 (0.6443)	0.000	1.000

**Note:** probability values in (), ** represent significance level at 5%.

#### 4.3.3 Reduced-form vector autoregressive

The VAR results for developed countries depict the reverse causality between financial inclusion and inflation rate [5, 6, 29]. A higher financial inclusion causes the inflation rate to decrease and effective monetary policy [3]. The financial inclusion and inflation rate results are presented in Table 11, which depicts an inverse relation between them in developing countries. However, at first lag, both are statistically insignificant. This may be because a lower degree of financial inclusion prevails in under-developed countries that hardly impact inflation. Out of controlled variables, economic growth has a significant impact on financial inclusion in developed countries. Inflation, money supply, and economic growth have a significant impact in developed countries (Table 7).

**Table 11:** Reduced-form VAR under-developed countries.

	FII	INF	ER	IR	MS	EG
**FII(-1)**	0.9159* [53.728]	‒0.1457 [‒0.782]	12.800** [2.058]	0.1604*** [1.738]	‒0.0516 [‒0.378]	‒0.3259* [‒2.553]
**INF(-1)**	‒0.0019 [‒0.467]	0.5730* [12.942]	‒0.2664 [‒0.180]	0.0745* [3.399]	‒0.0659** [‒2.033]	‒0.1006* [‒3.319]
**ER(-1)**	3.2306 [0.311]	‒7.8305 [‒0.689]	1.0360* [273.651]	1.2405 [0.221]	‒0.0002** [‒2.109]	0.0001 [1.291]
**IR(-1)**	0.0035 [1.263]	0.0397 [1.318]	0.8115 [0.809]	0.9727* [65.289]	0.0022 [0.099]	‒0.0058 [‒0.281]
**MS(-1)**	‒0.0002 [‒0.249]	‒0.0114 [‒1.209]	‒0.0288 [‒0.091]	‒0.0040 [‒0.856]	0.9856* 142.427]	‒0.0091 [‒1.404]
**EG(-1)**	0.0026 [0.419]	0.0072 [0.107]	0.3925 [0.175]	0.0737* [2.216]	0.0854*** [1.736]	0.3189* [6.930]
**R-squared**	0.882	0.358	0.995	0.936	0.985	0.182
**Adj. R-squared**	0.881	0.349	0.995	0.935	0.985	0.170

**Note:** t-Statistics in], *, **, *** represent significance level at 1%, 5% and 10%

#### 4.3.4 Impulse response functions

The impulse response function of developed countries is shown in (Fig 1(a)). The structural shock in inflation generates fluctuation in financial inclusion. The shock in inflation causes financial inclusion to decrease (panel a), and the response dies over time. Inflation’s negative response to the shock in financial inclusion (panel b) confirms the bidirectional causality.

**Fig 2:**
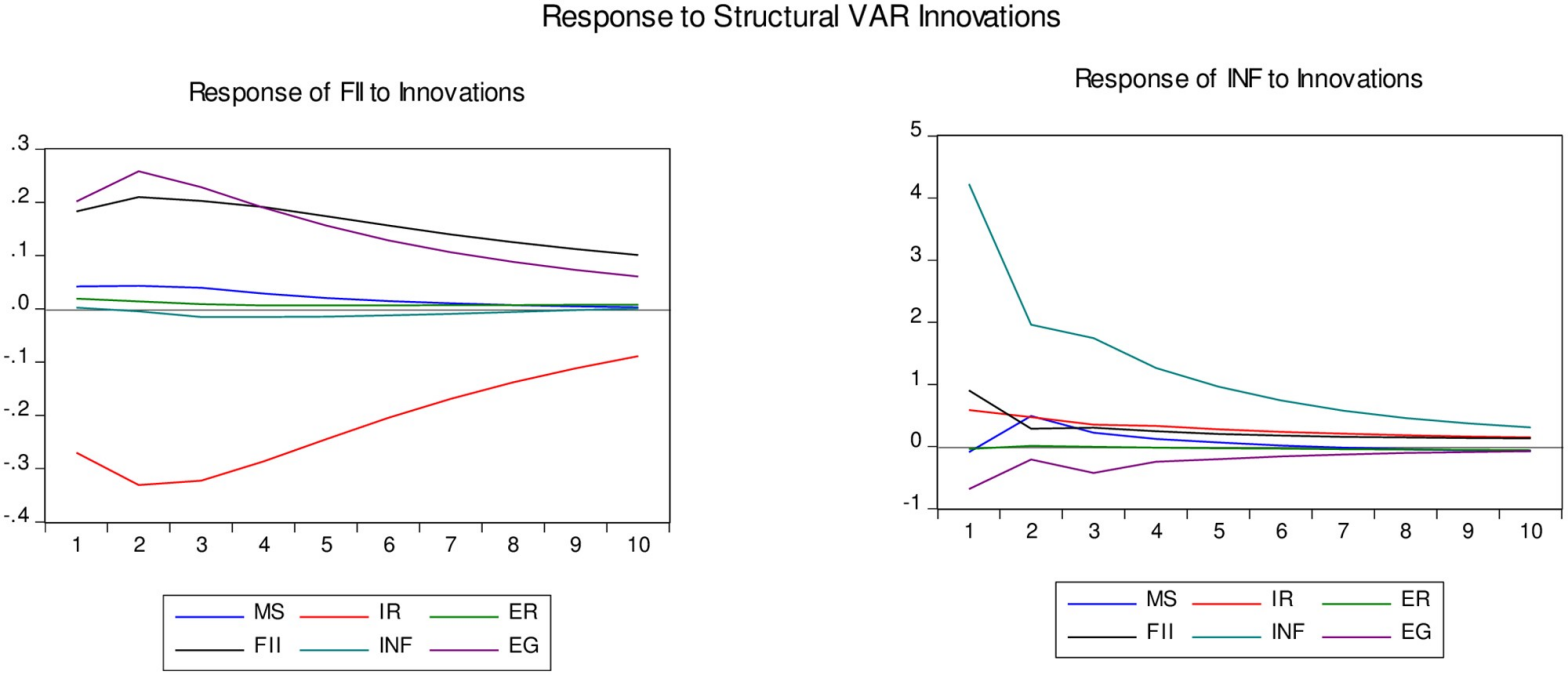
Impulse response of under-developed countries.

Fig 2 shows the results of IRFs in the case of under-developed countries. The shock in inflation creates a slight fluctuation in financial inclusion, but the response immediately dies off (panel a). Likewise, any structural shock in financial inclusion does not create strong fluctuations in inflation (panel b). The graph supports the earlier results, i.e., VAR and SVAR. Financial inclusion response negatively to the shock in interest rate initially and perishes gradually with time.

## 5. Conclusion and policy recommendations

The study examined the emergence of a link between the effectiveness of monetary policy and financial inclusion in developed and developing countries. The study employed a panel structural vector autoregressive technique on the data set from 2004 to 2018. Financial inclusion is quantified using three dimensions: access, utilisation, and barriers, with inflation serving as a proxy for monetary policy effectiveness. The SVAR results for developed and developing countries are identical in all models, demonstrating that financial inclusion and monetary policy effectiveness do not have a corresponding effect. However, the reduced-form VAR results indicate that financial inclusion and monetary policy effectiveness are inversely related in developed countries. Increased financial inclusion reduces inflation, which improves the effectiveness of monetary policy, and vice versa, an effective monetary policy increases financial inclusion. The results indicate that only the lag of inflation has a significant effect on financial inclusion in underdeveloped countries, implying that a higher level of financial inclusion results in more effective monetary policy in these countries. The evidence from developed countries suggests that greater financial inclusion enables more effective monetary policy, while effective monetary policy increases financial inclusion.

Financial inclusion policies should be developed with significant barriers to inclusion in mind, including distance, documentation, affordability, and trust. Various underdeveloped countries implement numerous policies to increase financial inclusion, but a significant gap persists. This study was limited to the data from specific developed and developing countries. Future research can be initiated with moderating role of financial development and digital finance to identify the relationship between financial inclusion and monetary policy.

## Supporting information

S1 Dataset (XLSX)

## References

[1] Publications, W.B., *The World Bank annual report 2013*. 2013: World Bank Publications.

[2] Mishkin, F.S., *The economics of money*, *banking*, *and financial markets*. 2007: Pearson education.

[3] Mbutor M.O. and Uba I.A., The impact of financial inclusion on monetary policy in Nigeria. Journal of Economics and International Finance, 2013. 5(8): p. 318–326.

[4] Lapukeni A.F., The impact of financial inclusion on monetary policy effectiveness: the case of Malawi. International Journal of Monetary Economics and Finance, 2015. 8(4): p. 360–384.

[5] Lenka S.K. and Bairwa A.K., Does financial inclusion affect monetary policy in SAARC countries? Cogent Economics & Finance, 2016. 4(1): p. 1127011.

[6] Anarfo E.B., et al., Monetary policy and financial inclusion in sub-Sahara Africa: a panel VAR approach. Journal of African Business, 2019. 20(4): p. 549–572.

[7] Ma Y. and Lin X., Financial development and the effectiveness of monetary policy. Journal of banking & Finance, 2016. 68: p. 1–11.

[8] Evans O., The effectiveness of monetary policy in Africa: modeling the impact of financial inclusion. Iranian Economic Review, 2016. 20(3): p. 327–337.

[9] Khan, H., *Financial inclusion and financial stability*: *are they two sides of the same coin*. Address by Shri HR Khan, Deputy Governor of the Reserve Bank of India, at BANCON, 2011.

[10] Mehrotra, A.N. and J. Yetman, *Financial inclusion and optimal monetary policy*. 2014.

[11] Mishkin F.S., Symposium on the monetary transmission mechanism. Journal of Economic perspectives, 1995. 9(4): p. 3–10.

[12] Bernanke B.S. and Gertler M., Inside the black box: the credit channel of monetary policy transmission. Journal of Economic perspectives, 1995. 9(4): p. 27–48.

[13] Khan H.H., Ahmad R.B., and Gee C.S., Bank competition and monetary policy transmission through the bank lending channel: Evidence from ASEAN. International Review of Economics & Finance, 2016. 44: p. 19–39.

[14] Hernando I., The credit channel in the transmission of monetary policy: the case of Spain. Topics in Monetary Policy Modelling, 1998: p. 257–275.

[15] Agha A.I., et al., Transmission mechanism of monetary policy in Pakistan. SBP-Research Bulletin, 2005. 1(1): p. 1–23.

[16] Brunnermeier M.K. and Sannikov Y., A macroeconomic model with a financial sector. American Economic Review, 2014. 104(2): p. 379–421.

[17] Abuka C., et al., Monetary policy and bank lending in developing countries: Loan applications, rates, and real effects. Journal of Development Economics, 2019. 139: p. 185–202.

[18] Sarma, M., *Index of financial inclusion (No*. *22259; Finance Working Papers)*. East Asian Bureau of Economic Research, 2008.

[19] Hannig, A. and S. Jansen, *Financial inclusion and financial stability*: *Current policy issues*. 2010.

[20] Demirguc-Kunt, A., et al., *The Global Findex Database 2017*: *Measuring financial inclusion and the fintech revolution*. 2018: World Bank Publications.

[21] Kim D.-W., Yu J.-S., and Hassan M.K., Financial inclusion and economic growth in OIC countries. Research in International Business and Finance, 2018. 43: p. 1–14.

[22] Jalil A. and Ma Y., Financial development and economic growth: time series evidence from Pakistan and China. Journal of Economic Cooperation, 2008. 29(2): p. 29–68.

[23] Chinoda, T. and F. Kwenda, *Do mobile phones*, *economic growth*, *bank competition and stability matter for financial inclusion in Africa*? Cogent Economics & Finance, 2019.

[24] Mugume, A., *Monetary transmission mechanisms in Uganda*. Editorial Board, 2009.

[25] Montiel, P.J., *The monetary transmission mechanism in Uganda*. 2013: International Growth Center, London School of Economics and Political Science.

[26] Mehrotra, A.N. and J. Yetman, *Financial inclusion-issues for central banks*. BIS Quarterly Review March, 2015.

[27] Mehrotra, A. and G. Nadhanael, *Financial inclusion and monetary policy in emerging Asia*, in *Financial inclusion in Asia*. 2016, Springer. p. 93–127.

[28] Hung, B.D., *Financial Inclusion And The Effectiveness Of Monetary Policy In Vietnam*: *An Empirical Analysis*. Division of Economic. Banking Academy. Hanoy, 2016.

[29] Brownbridge M., et al., The impact of Financial Inclusion on the interest rate channel of the monetary policy transmission mechanism. Bank of Uganda, Working Paper Series, 2017(05).

[30] Seth R. and Kalyanaraman V., Effect of financial development on the transmission of monetary policy. 2017. Policy. Theoretical Economics Letters, 7(1), 795–813.

[31] Huong N.T.T., The impact of financial inclusion on monetary policy: A case study in Vietnam. Journal of Economics and Development, 2018. 20(2): p. 5–22.

[32] Yetman, J., *Adapting monetary policy to increasing financial inclusion*. IFC Bulletins chapters, 2018. 47.

[33] Ma Y., Financial Development, Financial Structure, and the Growth Effect of Monetary Policy: International Evidence. Global Economic Review, 2018. 47(4): p. 395–418.

[34] Yin X., et al., The sustainable development of financial inclusion: how can monetary policy and economic fundamental interact with it effectively? Sustainability, 2019. 11(9): p. 2524.

[35] Carranza, L., J.E. Galdón-Sánchez, and J. Gómez Biscarri, *Financial development and the asymmetry of monetary policy*. SSRN 895163, 2006.

[36] Cámara N. and Tuesta D., Measuring financial inclusion: A muldimensional index. BBVA Research Paper, 2014(14/26).

[37] Enders, W., *Applied econometric time series fourth edition*. 2015, Wiley.

[38] Gujarati, D.N., *Econometrics by example*. Vol. 1. 2011: Palgrave Macmillan New York.

[39] Sims, C.A., *Macroeconomics and reality*. Econometrica: journal of the Econometric Society, 1980: p. 1–48.

